# A short-cut methodology for the spatial assessment of the biochemical river quality

**DOI:** 10.1007/s10661-024-12520-6

**Published:** 2024-03-21

**Authors:** P. Di Fluri, V. Di Talia, G. Antonioni, A. Domeneghetti

**Affiliations:** Department of Civil, Chemical, Environmental and Materials Engineering, Alma Mater Studiorum – University of Bologna, Bologna, Italy

**Keywords:** Water framework directive, Water quality, Biochemical quality index, Environmental pressure

## Abstract

The deterioration of superficial water quality is a significant concern in water management. Currently, most European rivers do not achieve qualitative standards defined by Directive 2000/60/EC (Water Framework Directive, WFD), while the health status of many surface water bodies remains unknown. Within this context, we propose a new methodology to perform a semi-quantitative analysis of the pressure state of a river, starting from easily accessible data related to anthropic activities. The proposed approach aims to address the endemic scarcity of monitoring records. This study proposes a procedure to (i) evaluate the relative pressure of different human activities, (ii) identify allocation points of different pollutant sources along the river using a raster-based approach, and (iii) determine a spatial biochemical water quality index. The developed index expresses the overall biochemical state of surface water induced by pollutant sources that may simultaneously impact a single river segment. This includes establishments under the so-called Seveso Directive, activities subjected to the IPPC-IED discipline, wastewater treatment plants, and contaminated sites. The methodology has been tested over three rivers in Northern Italy, each exposed to different industrial and anthropogenic pressures: Reno, Enza, and Parma. A comparison with monitored data yielded convincing results, proving the consistency of the proposed index in reproducing the spatial variability of the river water quality. While additional investigations are necessary, the developed methodology can serve as a valuable tool to support decision-making processes and predictive studies in areas lacking or having limited water quality monitoring data.

## Introduction

Adequate and high-quality water is essential for the sustainable development of human society (Uddin et al., 2021). However, anthropogenic activity can strongly modify the natural equilibrium of the freshwater ecosystem, leading to a deterioration of water quality (Khatri & Tyagi, 2015; Peters & Meybeck, 2000; Vigiak et al., 2023), which can be exacerbated or mitigated, by climate change (Paerl et al., 2020). In such a context, the target of the EU’s water policy is to promote measures and solutions to ensure good-quality water for people and the environment. The Water Framework Directive (WFD) (EU directive 2000/60/EC, 2000) established a framework for the assessment, management, protection, and improvement of the status of water bodies across the EU. In particular, the WFD requires Member State to assess the status and pressures of water bodies with a River Basin Management Plan – RBMP (European Commission, 2012) and to monitor, where necessary, the status of water bodies defining a Programme of Measures (PoMs) that must be revised every six years (Skoulikaris & Zafirakou, 2019). Since December 2015, EU Member States have been publishing the second RBMPs to report on (i) the status of EU waters, (ii) the pressures causing less than good status, and (iii) the progress achieved during the RBMP cycle.

Unfortunately, recent reports indicate that a significant amount of European surface water bodies do not attain a good ecological status (EEA, 2012, 2018a). More specifically, 60% of the surface water bodies fail to achieve the target of good ecological condition (Nikolaidis et al., 2022). Furthermore, the monitoring system across the EU appears to be critical: Datasets are not spatially and temporally homogeneous, often resulting in representations that are not reflective of the actual quality status of the water body (Irvine, 2004), while, in some cases, the monitoring data of chemical and biological parameters are reported for different sampling periods (Malaj et al., 2014).

**Fig. 1:**
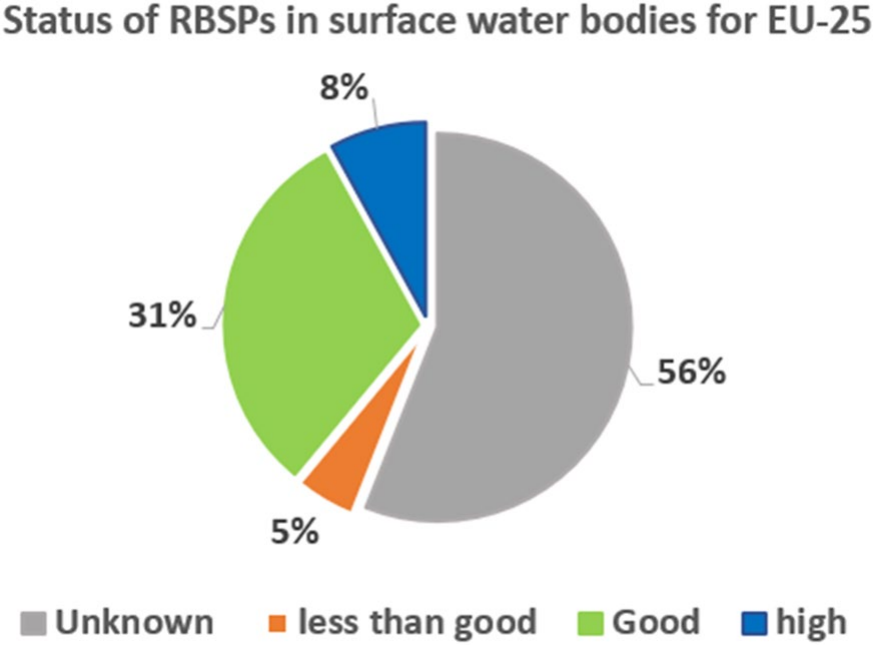
European watercourse status based on monitoring data from EU-25 (adapted from EEA, 2018a, b)

The inadequacy (i.e., limited spatial and temporal coverage) of monitoring networks results in a lack of knowledge regarding the health status of a vast portion of the water bodies. Figure 1 refers to EU-25 countries (EEA, 2018b) and shows that the status of the watercourses is unknown in 56% of the cases. Although this data shortage could potentially be attributed to the defaulting of some EU Members in communicating reports on fluvial status, it highlights the need to strengthen monitoring capabilities. This represents a key limitation, as the absence of monitoring data hampers the identification and quantification of pressures on the water system, as well as the evaluation of their correlation with water quality (Grizzetti et al., 2017). As a consequence, designing efficient PoMs becomes one of the most challenging aspects of the WFD.

Many water bodies in Europe face heterogeneous pressures. These pressures often act simultaneously and affect the good functioning of ecosystems, contribute to biodiversity loss, and threaten the valuable benefits that water brings to society and the economy (Schinegger et al., 2012). Thus, assessing and prioritizing their impacts is essential to drive effective mitigation measures (Jackson et al., 2018; Navarro-Ortega et al., 2015). The scientific literature reports many studies that investigate the relationship between water quality deterioration and anthropogenic pollution, identifying categories and types of activity that primarily contribute to the deterioration of river quality (Akhtar et al., 2021; Ma et al., 2020; Qin et al., 2014). Industrial effluents are the main anthropogenic pressure for the aquatic ecosystem, discharging heavy metals, pesticides, chemicals, and petrochemicals compounds into the receiving water body (Adewumi et al., 2011).

Available methods to assess ecological status differ in their capacity to consider the anthropogenic pressures and assessment criteria (Poikane et al., 2019, 2020; Santos et al., 2021). The Water Quality Index is one of the most popular indexes providing a quantitative assessment of watercourse degradation due to anthropogenic pressures. In particular, it adopts aggregation techniques to convert extensive datasets on water quality into a single value or index (Poonam et al., 2013; Uddin et al., 2021). In a similar fashion, a recent study by Mirauda et al. (2021) proposes a methodology to assess the resilience of the river to pollution from urban and industrial discharges. However, these indices rely on a large amount of water quality and flow data, which often are scarce or absent. The necessity for monitoring data presents a constraint on the extensive application of such indexes, rendering them unsuitable for addressing water quality assessment in unmonitored watercourses. Recently, Arrighi et al. (2018) and Yao et al. (2015) proposed empirical methods for the identification and classification of risk source typologies for watercourses, which do not refer to monitoring data and present alternative ways to depict the overall human pressure over river and natural resources.

Nevertheless, they fall short in addressing significant issues, such as the identification of the locations where human pressures driven manifest the recognition of the river sections most profoundly impacted. Moreover, a comprehensive understanding of the contribution of each anthropogenic activity to the overall chemical load remains unknown. In response to these gaps, we propose a methodological framework to assess freshwater quality at a basin and sub-basin level based on the spatial distribution of the anthropogenic pressures.

The novelty of the proposed methodology stems from its capability to assess the pressures exerted by anthropogenic activities utilizing readily available data that characterize the polluting source affecting the watercourse. The presented methodology is aimed at compensating for the lack of water quality data in poorly or not-monitored rivers, assisting and supplementing conventional monitoring efforts, and supporting the identification of proper mitigation measures.

The methodology is presented in the “Methods and materials” section, which also provides details on assumptions and data required for its implementation. The methodology has been applied to three case studies. The validation and the obtained results are reported in “Results and discussion” section. Strengths and limitations of the current approach are summarized in the “Conclusions” section.

## Methods and materials

**Fig. 2:**
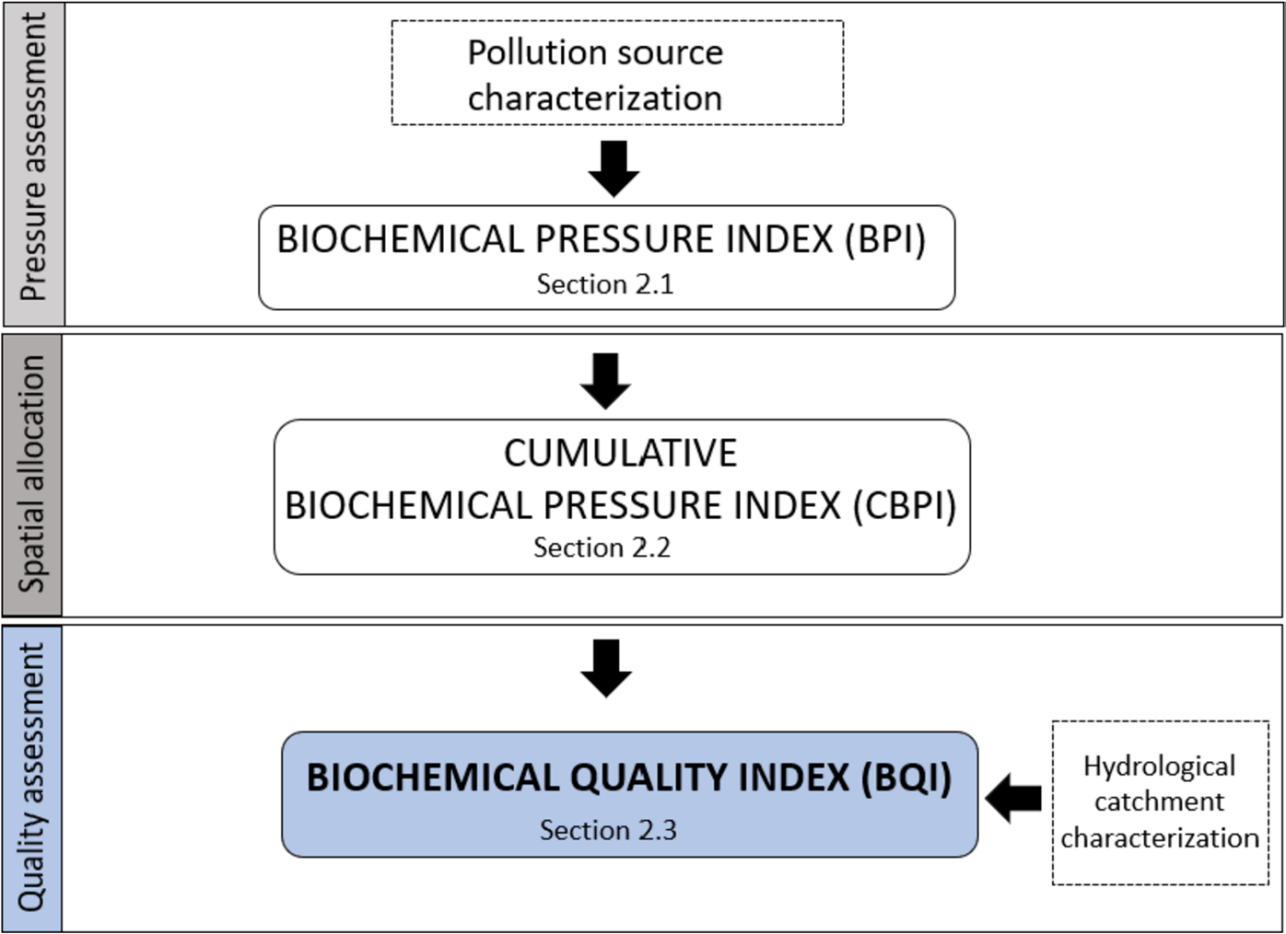
Flow chart representing the methodology

The study’s main scope is the definition of a short-cut multidisciplinary methodology for the spatial assessment of a pressure index that can be adopted to evaluate the biochemical river quality starting from easily accessible data. The methodology consists of three main steps, as schematically represented in Fig. 2:

i) identification and classification of anthropogenic pollution sources by assessing an expert-based and data-driven index (“pressure assessment” phase);
ii) spatial allocation of pollution sources and identification of their allocation points along the river (“spatial allocation” phase);
iii) spatial water quality assessment based on hydrological characteristics (“Quality assessment” phase).

Pressure assessment is addressed in the “Biochemical Pressure Index” section, while spatial allocation and quality assessment are addressed in the “Cumulative Biochemical Pressure Index” and “The Biochemical Quality Index” sections, respectively.

### The Biochemical Pressure Index (BPI)

A semi-quantitative Biochemical Pressure Index (BPI) is proposed to evaluate the pressure on surface water bodies originating from selected anthropic activities. The overall anthropogenic pressure on rivers includes point and diffuse pollutant sources. It is necessary to point out that addressing diffuse pollution requires an accurate and targeted monitoring program. In fact, diffuse sources are mainly attributed to agricultural activities that are responsible for excessive emission of nutrients, such as nitrate and phosphorus (Grizzetti et al., 2017; Nikolaidis et al., 2022). Though significant in terms of nutrients, agricultural load in total oxygen demand is usually not relevant, and its estimation is not straightforward across large scales. In fact, agricultural activities are typically seasonal: The pressures on water bodies can be higher during certain periods when activities are concentrated, such as spring planting or fall fertilization. This also implies that the pollutant load deriving from these activities may vary in time and space due, for example, to crop rotation, growing techniques and practices, etc., and not be significant when considering the average annual pollutant load, as in the present study. Also, available data describing such practices is poor and uncertain. Based on these considerations, diffuse pollution sources from agriculture are neglected in the presented approach.

Besides that, some diffuse sources are indirectly considered (namely contaminated sites) since they are likely to contribute to some extent to the overall oxygen demand. Point source selection has been driven based on expert judgment and literature outcomes (i.e., Poikane et al., 2019, 2020; Prakash & Verma, 2022). Among industrial facilities, establishments under Directive 2012/18/EU (so-called Seveso Directive) and activities subjected to Directive 2010/75/EU (IPPC-IED) can be considered the most relevant as they normally produce and store large amounts of substances harmful to the aquatic environment (Kanu & Achi, 2011; Shafiei Moghaddam et al., 2023). It is noteworthy to note that for these activities, it is compulsory to report typologies and quantities of hazardous substances stored and eventual discharges.

Moreover, wastewater treatment plants (WWTPs) are often sources of nutrients for receiving water bodies, attributable to particular hydrological regimes or failures of control systems (Carey & Migliaccio, 2009; Preisner, 2020; Su et al., 2021). The WWTP dataset was skimmed by neglecting septic and Imhoff tanks and considering only activated sludge plants, as they discharge into water bodies or sewers. Finally, contaminated sites (CS) can also significantly contribute to the pollutant load on surface waters due to polluted run-off water from former industrial and urban areas (Chaudhry, 2017; Gnecco et al., 2005; Legret & Pagotto, 1999). Considering these selected categories of anthropogenic pollution sources, the BPI aims to straightforwardly quantify the pressure derived from the discharges of these activities, considering each release as an individual source. The BPI is defined as the product of the values attributed to five parameters (Eq. (1)):

1 $$BPI = D\cdot T\cdot F\cdot H\cdot S$$

where parameters type and description are listed in Table 1, while categories and scores are reported in Table 2. Each parameter considers distinct aspects that can affect the magnitude of each contamination source: The higher the assigned value, the higher the pressure that the discharge can exert on the water body. Parameter *D* represents the type of discharge in terms of its expected impact on surface water quality; thus, it ranges from less hazardous uncontaminated run-off water discharges to discharges into the aquatic environment of industrial wastewater. Prior to reaching the receiving water body, wastewater can be treated or not (clearly also depending on local in-force legislation and the nature of contaminants), and this is considered in the second parameter of the index *T*. Then wastewater, treated or not, could be discharged directly into the river or collected and sent to the public sewer as considered by parameter *F*. The nature or typology of contaminants (strictly related to the anthropic activity) and especially their potential hazard on the aquatic environment is represented by parameter *H*. Finally, the wastewater flow rate is quantified by means of parameter *S*.

**Table 1:** Parameters adopted for the calculation of the BPI

Parameter	Description
Type of discharge (D)	Identification of the discharge type (see Table 2 for details)
Treatment (T)	Presence or absence of physical, chemical, and/or biological treatments before the discharge
Fate (F)	Fate of the discharge (receiving element)
Presence of hazardous substances toxic to the aquatic environment (H)	Indication and quantification of the presence in the discharge of hazardous substances toxic to the aquatic environment according to the Classification, Labelling and Packaging (CLP) Regulation ((EC) No 1272/2008)
Size (S)	Discharge rate parameter

^a^Documentation retrieved consulting the website: http://ippc-aia.arpa.emr.it/ippc-aia/Homepage.aspx

^a^Database accessed at the link: https://datacatalog.regione.emilia-romagna.it/catalogCTA/dataset/depuratori-della-regione-emilia-romagna-1506530997461-718

^a^Database accessed at the link: https://datacatalog.regione.emilia-romagna.it/catalogCTA/dataset/elenco-dei-siti-contaminati-della-regione-emilia-romagna-1523632340215-121/resource/cf8b31d0-8862-4579-95ef-af2e13bb229d]

**Table 2:** Severity scales of BPI parameters

Parameter	Attribute	Value
Type of discharge (D)	Uncontaminated surface run-off water	1
	Domestic wastewater	2
	Contaminated surface run-off water and washing wastewater	3
	Industrial wastewater	4
Treatment (T)	Yes	0.5
	No	1
Fate (F)	Public sewer	0.5
	Surface water body	1
Presence of hazardous substances toxic to the aquatic environment (H)	Absence. This value is also attributed to CS when the remediation is concluded	1
	Presence, regardless of quantity, for IPPC-IED activities and to a lesser extent of the lower-tier requirements (Directive 2012/18/EU) for Seveso activities*; this value is also attributed to CS when the remediation is not concluded	1.5
	Presence in quantities between the lower-tier and upper-tier requirements (Directive 2012/18/EU) for Seveso activities	2
	Presence in quantities greater than the upper-tier requirements (Directive 2012/18/EU) for Seveso activities	3
Size (S)**	0–10^3^ m^3^/year	0.5
	10^3^–10^4^ m^3^/year	1
	10^4^–10^5^ m^3^/year	1.5
	10^5^–10^6^ m^3^/year	2
	10^6^–10^7^ m^3^/year	2.5
	> 10^7^ m^3^/year	3

*The reference for the rating’s attribution is Directive 2012/18/EU (Annex I, Part 1, section E). For Seveso and IPCC-IED activities, the presence of hazardous substances toxic to the aquatic environment is evaluated only in discharges of types 3 and 4

**Point source relevance based on m^3^ discharged/year. S = 0.5 in case the value is not explicitly declared (typical for small plants)

The values assigned to each parameter (as shown in Table 2) have been initially attributed based on expert judgment in order not to over- or under-estimate its effect in the calculation of the final index. Afterward, they have been checked and refined thanks to an ex-post analysis, as described in the “Parma River” section.

Concerning parameter *D*, industrial wastewater is naturally evaluated as more dangerous (rating 4), followed by contaminated surface run-off water and washing wastewater (rating 3), while domestic wastewater (rating 2) and uncontaminated surface run-off water (rating 1) can be readily classified as the discharge typologies with a potentially less significant impact on surface water. These typologies have been selected based on the wastewater classification taken from the Directive 91/271/EEC. They are required to be reported in the IPPC-IED documentation for the characterization of discharges, making them particularly suitable for the present analysis.

Regarding parameter *T*, a value of 0.5 is given when the wastewater is treated before the discharge and 1 when it is not, as this implies a lower pollution load into surface water bodies.

A similar approach has been adopted for parameter *F*: 0.5 for discharges into the public sewer (this situation implicitly includes further treatment) and 1 for releases directly into surface water bodies. These last two parameters can be both considered as barriers to water pollution.

Values assigned to parameter *H* strictly depend on the substances produced by different activities that can be found in wastewater and that can be extremely heterogeneous. Thus, its assessment depends on the typology of the anthropic activities. For plants under Directive 2012/18/EU, in Annex I, Part 1, section E (“Seveso” activities), threshold values for hazardous substances are given, including substances that may have a negative impact on the aquatic organisms or environment (R50 to R53 phrases in Globally Harmonized System of Classification and Labelling of Chemicals (GHS Rev. 9, 2021)). These substances can be considered as the most hazardous among those potentially present in wastewater; thus, in this case, a value of 3 or 2 is assigned to parameter *H*, depending on whether they are above the upper threshold reported in Directive 2012/18/EU or between the lower and the upper threshold, respectively (see Table 2). This conservative assumption is that such plants may produce wastewater containing chemicals that may still increase the oxygen demand of the river, even if some substances are not classified as harmful or toxic to the aquatic environment. For IPCC-IED activities, the presence of hazardous substances toxic to the aquatic environment is considered only for discharges of typologies 3 and 4 (parameter D), according to 2010/75/EU. In this case, it has been assumed that this category of activities is likely to produce wastewater with significant concentrations of substances that can be dangerous for surface water.

In the case of contaminated sites, where a variety of different and hazardous substances may be present, the primary discrimination factor is whether remediation has been completed. If this is the case, a score of 1 is assigned; otherwise, a score of 1.5 is given, indicating that hazardous substances may still present in a non-negligible concentration into the soil, with the potential risk of migration to surface water because of runoff and/or rain. Finally, for WWTPs, a value of 1 is assigned since they are supposed to deliver treated water into the water body.

Eventually, a parameter suitable for considering the amount of discharged water has been introduced (*S* in Eq. (1)) in order to correctly take into account the size of the pollution source: starting from 1000 m^3^ discharged/year, a 0.5 point increase per each order-of-magnitude increase in the discharged flowrate expressed in m^3^/year has been assumed (see Table 2 for details). The first group (0–1000 m^3^ discharged/year) also includes the discharge rates that are not reported nor available since it has been verified that the flow rate is reported only for significant discharges (typically above 1000 m^3^/year).

### Cumulative Biochemical Pressure Index (CBPI)

According to the methodological framework (see Fig. 2), once the BPI is defined for each pollution source, the evaluation proceeds considering their spatial distribution on the river basin and their allocations along the river network.

**Fig. 3:**
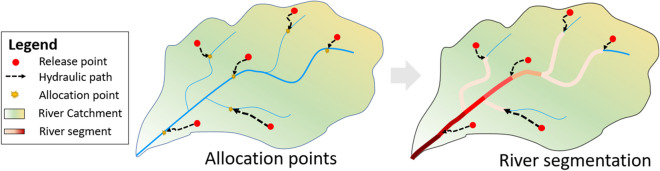
Schematization of the procedure for BPI spatial allocation

The analysis comprises the following steps, which are also schematized in Fig. 3:

1. DEM-based (digital elevation model) identification of hydraulic paths for each release point and river segmentation; the main river network is split into a new segment every time a new allocation reaches the main watercourse.
2. Assessment of the spatial dynamic of the biochemical pressure along the river network by estimating the Cumulative Biochemical Pressure Index.

The hydraulic path is derived by means of a raster-based approach able to describe the draining direction starting from a source cell based on the maximum topographic gradient. This latter is estimated considering a flow direction generated using a standard D8 methodology (Garbrecht & Martz, 1997) applied to a pre-treated DEM to ensure the hydraulic continuity of the streams (Lehner et al., 2008) (see Fig. 3, left panel). Then, the main river network is split into a different segment every time there is a lateral inlet associated with a pollution source (Fig. 3, right panel).

The DEM-based delineation of the hydraulic path is common in hydrological applications. Nevertheless, the delineation of the draining direction is affected by uncertainty, which depends on several factors, such as DTM resolution (the finer, the better; Ariza-Villaverde et al., 2015) and DTM origin (i.e., satellite-based products may be affected by significant biases that vary in space in relation to land use or topography Moretti & Orlandini, 2023; Yamazaki et al., 2019). In addition, the reliability of this approach falls short when referring to plain areas, where the presence of urban (e.g., roads) and hydraulic (e.g., embankment) infrastructures may alter the identification of the hydraulic path.

The spatial assessment of the biochemical pressure considers two aspects that characterize transport in surface waters: (a) the cumulative effect due to increasing pollutant load moving downstream and (b) the auto-depurative effect associated with the dilution capacity of the river. With reference to the first aspect, moving from upstream toward downstream along the river, the Cumulative Biochemical Pressure index (CBPI) is calculated as the sum of all BPIs associated with hotspots that flow into the main river (Eq. (2)):

2 $$CBPI= \sum_{i}{BPI}_{i}$$

Based on its definition, it is worth noting that the CBPI is expected to increase along the river moving downstream (the red color bar in Fig. 3, right panel, depicts the expected spatial CBPI dynamic: the darker, the higher the pressure on the river segment).

### The Biochemical Quality Index (BQI)

The environmental quality of receiving water bodies depends on the load of contaminants discharged, as well as on the self-purification capacity of the water bodies, which includes several physico-chemical processes naturally occurring within the river, including simple dilution, dispersion, biodegradation, sedimentation, and adsorption. Proper evaluation of load distribution requires detailed and spatially varied discharge records along the river network, which are typically unavailable. Also, many other river data and parameters are not easily accessible or estimable, especially when assessing water quality for an entire basin using mechanistic models (Sharma & Kansal, 2013). Among these processes, dilution and dispersion play dominant roles in mitigating water quality degradation. Thus, to address the lack of data and parameters, the drainage area estimated for each river segment is used as a proxy for the expected river flow (Yang et al., 2019), which in turn acts as a dilution parameter for the pollution load expressed in terms of CBPI.

Based on this assumption, dividing the CBPI of a given river segment (CBPI_*i*_) with its draining surface (*A*_i_), we estimated the Biochemical Quality Index (BQI) as for Eq. (3):

3 $${BQI}_{i}=\frac{{CBPI}_{i}}{{A}_{i}}$$

**Fig. 4:**
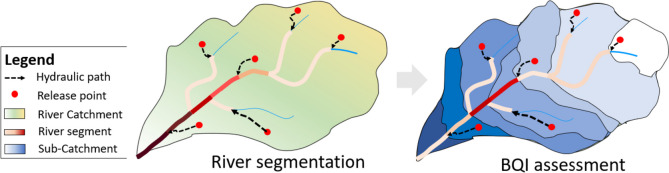
Schematic representation for drainage basins detection and BQI assessment

The delineation of the drainage basin for each river segment and the calculation of its extent have been performed using the GRASS GIS tool “*r.water.outlet*,” adopting a DEM with a planimetric resolution of 5 m. Figure 4 provides a schematic representation of the methodology implemented for CBPI (left panel) and BQI calculation (right panel).

The outcome is a spatially distributed index that estimates biochemical water quality caused by the anthropogenic pressure on freshwater based on the spatial distribution of pollution sources and the catchment features. The index indirectly takes into account dilution and all the complex self-purification processes occurring within river water (see the “Conclusions” section for additional discussion on that). The ability of the proposed index to capture all these features and provide an estimation of actual water quality will be verified in the “Results and discussion” section by comparing the BQI against the limited available measured COD data.

### Implementation data and assumptions

This section presents a brief guide for BPI estimation. All the relevant information concerning the pollution sources and their features has been collected from the available documentation. A brief overview of the documents consulted for the implementation of the case studies (“Results and discussion” section) per activity typology is presented in Table 3. The document names are reported in Italian and translated into English (in brackets) for clarity.

**Table 3:** Documentation consulted for the activity typology at stake

Activity typology	Documentation
Seveso	*Piano di Emergenza Esterna (*External emergency plan), *Autorizzazione Integrata Ambientale* (Integrated environmental authorization)^a^, *Autorizzazione Unica Ambientale* (single environmental authorization)^1^
IPPC-IED	*Autorizzazione Integrata Ambientale* (integrated environmental authorization)
WWTPs	*Depuratori della Regione Emilia-Romagna* (wastewater treatment plants of Emilia-Romagna region) dataset^a^
CS	*Elenco dei siti contaminati della Regione Emilia-Romagna GSI_1.1* (list of contaminated sites in the Emilia-Romagna region GSI_1.1) dataset^a^

Depending on the country or region of interest, official documentation and databases concerning these activities can be provided by competent authorities or be freely available to the public for the region of our interest (e.g., Emilia-Romagna).

The reference document for Seveso activities is *Piano di Emergenza Esterna*, which is available on the website of the competent Prefecture. This document enables evaluation of the presence of hazardous substances toxic to the aquatic environment and provides the information to attribute a value to parameter *H*. These activities are typically subjected also to the IPPC-IED discipline and, alternatively, to DPR n.59/2013. The related documentation (*Autorizzazione Integrata Ambientale* and *Autorizzazione Unica Ambientale*, respectively) usually provides the information necessary to attribute a value to parameters *D*, *T*, *F*, and *S*.

For IPCC-IED activities, the reference document is *Autorizzazione Integrata Ambientale*. In particular, the sections to address are the ones related to discharges and plant monitoring. Regarding parameter *H*, a value of 1.5 is to be given to the discharges in which the presence of hazardous substances toxic to the aquatic environment can be assumed, considering the primary type of activity carried out on the premises. As landfills are particularly relevant for this study due to the presence of a significant amount of hazardous liquid effluents, the size parameter of potentially contaminated discharges (types 3 and 4) is to be defined according to the m^3^/year of leachate produced.

Regarding wastewater treatment plants (WWTPs) and contaminated sites (CS), two databases extracted from the regional *Portale MinERva* have been used (see notes to Table 3 for more details). Reasonable assumptions are proposed to account in case of lack of required data: For both WWTPs and CS, the presence of a single discharge can be assumed if detailed information on the plant is lacking. Concerning WWTPs, the release can be considered assimilable to domestic wastewater; it is reasonable to assume that treatment is present, the fate of the discharge is presumed to be in the surface water body, and hazardous substances toxic to the aquatic environment are considered absent. In order to define the parameter size *S*, an equivalent flow rate can be calculated based on an average value, estimated from real data, of the volume flow rate treated or discharged and the population equivalents ratio (/PE). Usually, the population equivalent value is provided.

For CS, the discharge can be regarded as assimilable to industrial wastewater; it is reasonable to assume that treatment is present, and its destination is considered to be the surface water body. Concerning the *H* parameter, a value of 1 is attributed to remediated sites where remediation has been completed and 1.5 to sites where remediation is still to be performed or is ongoing. Finally, the hazard source is considered small.

Table 4 summarizes previous considerations and reports ranges of parameters and assumed values according to activity typology.

**Table 4:** Parameters values in relation to activity typology

	D	T	F	H	S
Seveso	[1–4]	[1–0.5]	[1–0.5]	[1.5–3]	[0.5–3]
IPPC-IED	[1–4]	[0.5–1]	[0.5–1]	1.5	[0.5–3]
WWTPs	2	0.5	1	1	[0.5–3]
CS	4	0.5	1	[1–1.5]	0.5

Starting from the information collected and the assumptions described, it is possible to assign a value to each parameter listed in Table 1, and thus, the BPI can be easily calculated for each source as the product of these values according to Eq. (1). Finally, from BPI, cumulative index (CBPI) and quality index (BQI) can be calculated according to the procedure described in the “Biochemical Pressure Index” section and Eqs. (2) and (3), respectively.

### Study areas

We tested the implementation of the proposed methodology referring to three case studies in the Emilia-Romagna region (Central-Northern Italy). The case studies have been selected because they present sufficient monitoring data, different watershed hydrological conditions, heterogeneity in the types of pollutant sources, and, hence, different levels of pressure on water bodies. For all case studies, information concerning pollution hotspots and their features has been collected from the available documentation (Table 3). Starting from this information, we calculated the biochemical indexes according to Eqs. (1)–(3). In addition, a validity check of the outcomes was possible due to freely available water quality data monitored at selected locations along the rivers.

#### Study area 1 – Reno River

The Reno River catchment (Central-Northern Italy) has an overall extent of 5040 km^2^. Originating from the Emilia-Romagna and Tuscan Apennines, it crosses the Pianura Padana plain before reaching the Adriatic Sea after a distance of 212 km.

**Fig. 5:**
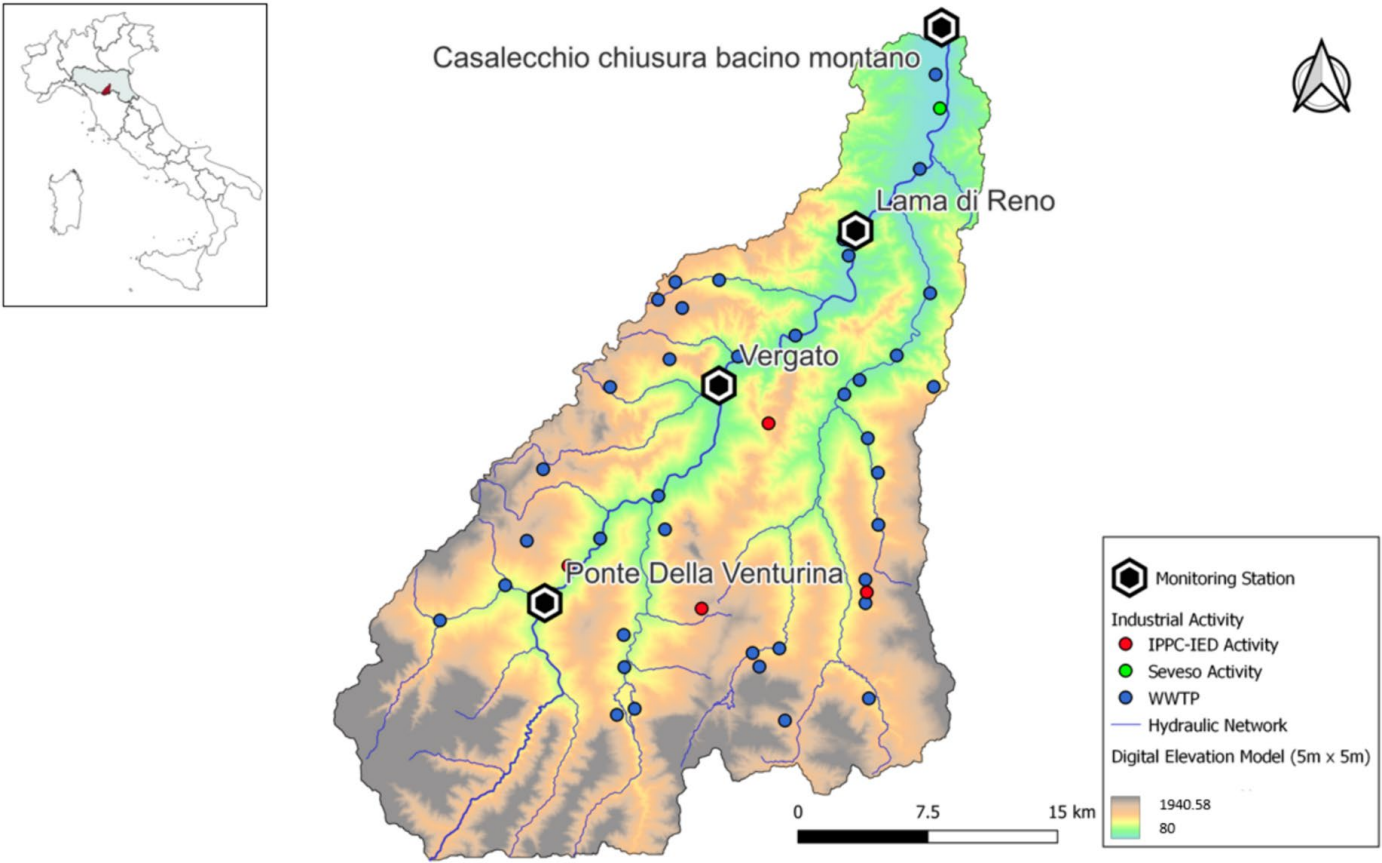
Reno River catchment: pollution points and main river network. Box (height-left corner) identifies the catchment location within the region (Emilia-Romagna; in gray) in Central-Northern Italy

The current configuration of the river network is the result of the countless modifications made since Roman times, with extensive remediation and hydraulic protection works that severely impacted the river network, particularly the downstream portion of the river. The reclamation of the surrounding flood-prone areas has led to a radical change in the river basin where the surface water, beyond the city of Casalecchio, flows within artificial embankments that carry the water to the Adriatic Sea. In this work, we refer to the river basin delimited at the monitoring station of Casalecchio Chiusa (see Fig. 5), which subtends a basin of 709 km^2^ (average and max elevation equal to 639 m and 1945 m a.s.l., respectively). Recorded mean daily discharge at Casalecchio (monitored since 1923) varies from 0.48 to 2200 m^3^/s, with a mean value of 17.9 m^3^/s.

According to the “Methods and materials” section, 46 pollution hotspots have been identified in the Reno catchment (Fig. 5): 1 Seveso plant, 4 IPPC-IED activities, and 41 WWTPs. Details on available monitoring data are reported in the “Monitoring data for validation” section.

#### Study area 2 – Enza River

**Fig. 6:**
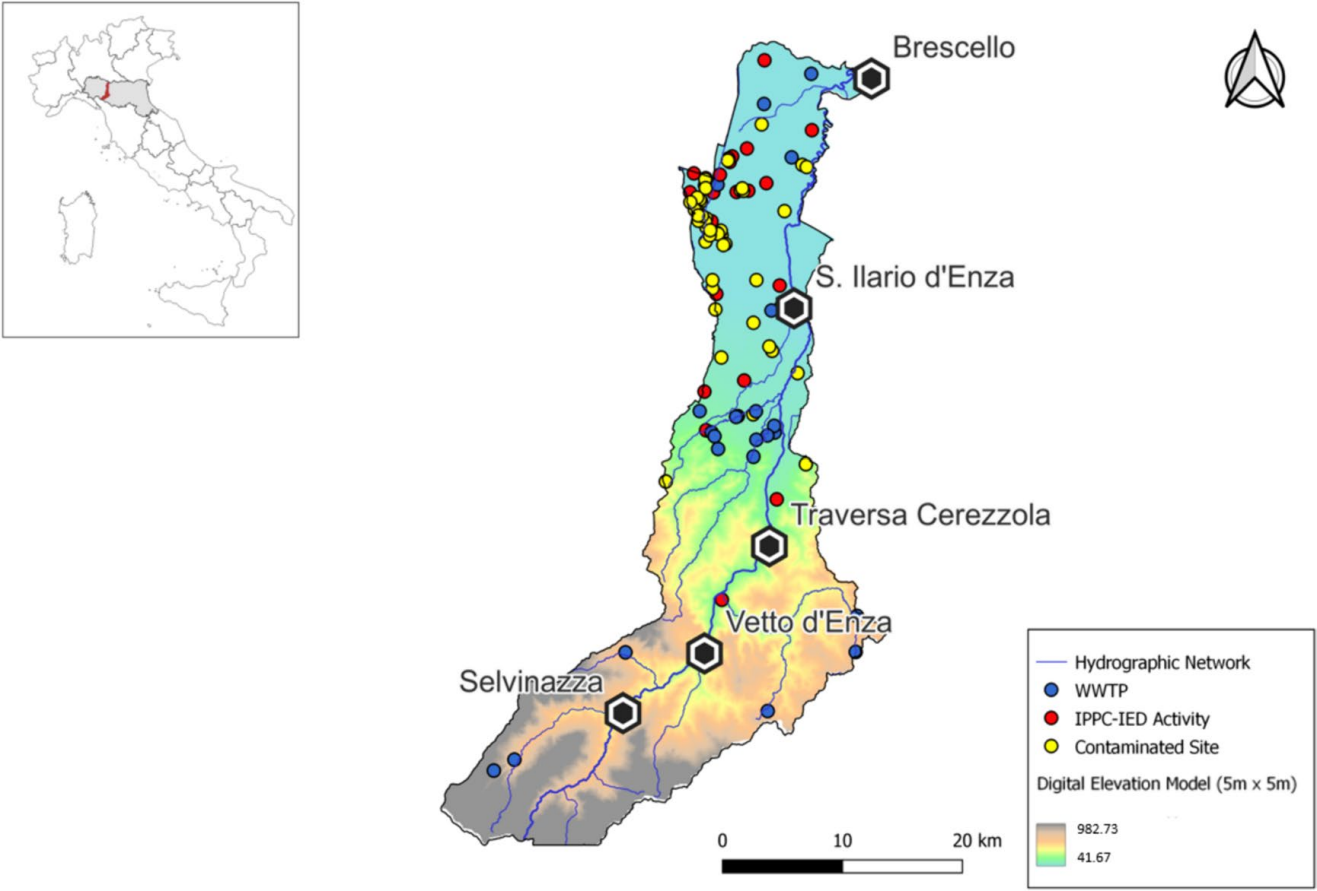
Enza River catchment: pollution points and main river network. Box (height-left corner) identifies the catchment location within the region (Emilia-Romagna; in gray) in Central-Northern Italy

The Enza River catchment (Central-Northern Italy) has a total area of 857 km^2^. The Enza River is a right tributary of the Po River. It originates in the Tuscan region, in the municipality of Comano, and demarcates the border between the provinces of Parma and Reggio Emilia from its entrance into Emilia-Romagna almost up to its mouth. We applied the methodology illustrated in the “Methods and materials” section to the 96 pollution hotspots identified in the Enza catchment (Fig. 6): 26 IPPC-IED, 24 WWTPs, and 46 CS.

The relevant information concerning these hotspots and their features has been collected from the available documentation (see Table 4).

#### Study area 3 – Parma River

**Fig. 7:**
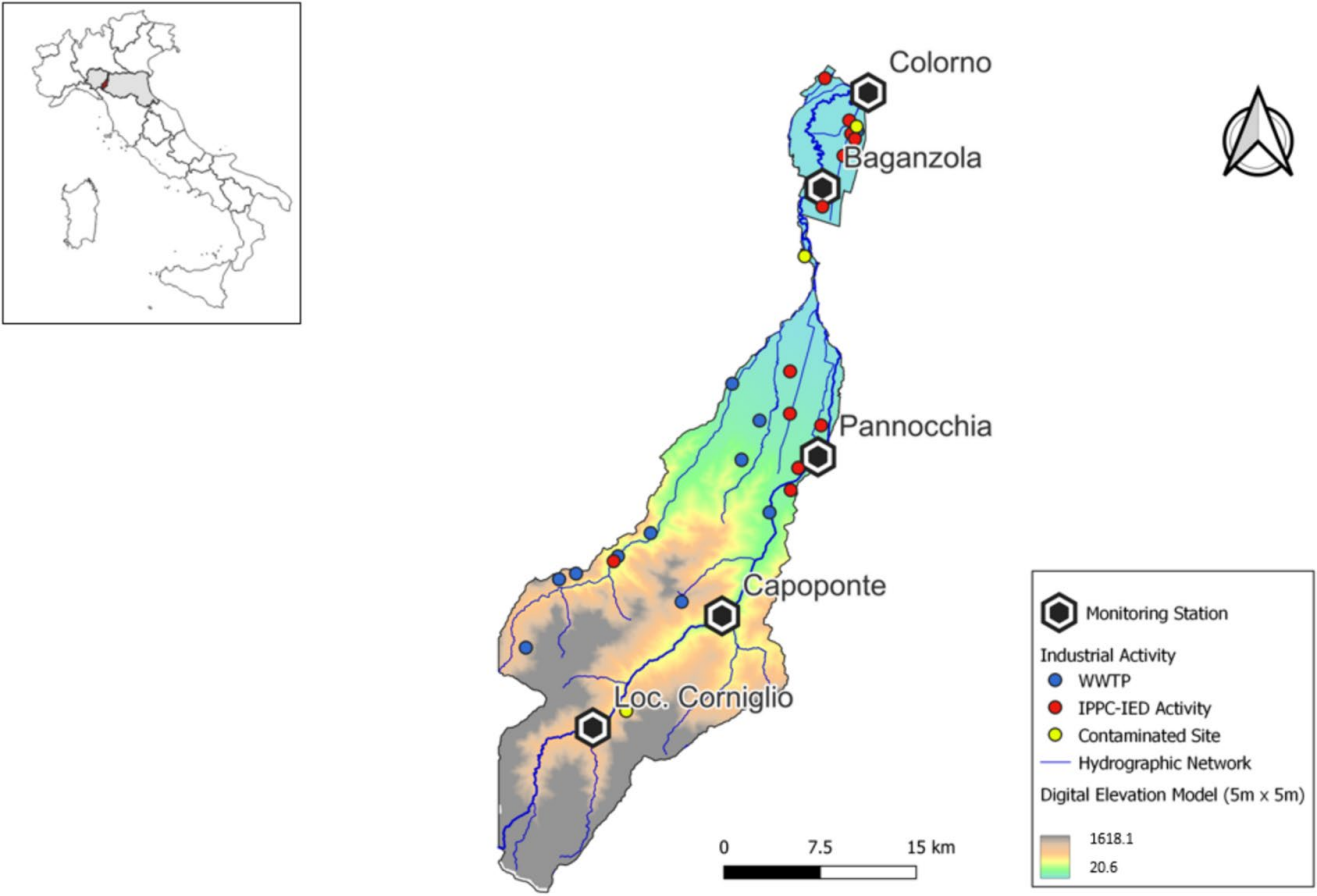
Parma River catchment: pollution points and main river network. Box (height-left corner) identifies the catchment location within the region (Emilia-Romagna; in gray) in Central-Northern Italy

The Parma is an Italian torrent of 92 km, the right tributary of the Po River, which develops entirely within the province of Parma, in Emilia-Romagna (Fig. 7). It has a catchment area of 608 km^2^. The methodology set out in the “Methods and materials” section was applied to the Parma basin, which contains 35 polluting sources, of which 13 are WWTPs, 8 IPPC-IED activities, and 14 contaminated sites.

### Monitoring data for validation

Data used for the validation has been retrieved from the ARPAE portal of the Emilia-Romagna region.^2^ The data relates to the monitoring stations belonging to the regional network and is considered for water quality assessments, according to WFD. This dataset involves recording values of general chemical parameters for the definition of biological status (e.g., nitrates, phosphorus, COD, and BOD), polycyclic aromatic hydrocarbons, metals, volatile organic compounds, pesticides, and contaminants from Table 1/A and 1/B of Decree 260/2010, the Italian transposition of the WFD, for the assessment of chemical status. Unfortunately, water quality data are not associated with river flow information. This prevents, at this stage, any investigations concerning the temporal water quality dynamics in relation to hydrological regimes (e.g., droughts or floods).

The observations cover the period from 2010 to 2020. However, the dataset is incomplete, with missing data that results in poor spatial (i.e., missing data for several monitoring stations, especially along the minor river network) and temporal (i.e., fragmented time series available at the monitoring stations) coverage. The reference COD value considered at a given gauging station for validation purposes is the annual average of available surveys (typically from 2 to 12 measures per year per gauging station). This approach is in line with the one adopted to assess the ecological status of superficial water bodies in Italy, which refers to three-year averaged values. Also, this enabled us to overcome the lack of monitoring data that occasionally occurs at a given gauging station.

In particular, the validity check has been performed considering 2017 as the reference year, chosen in consideration of the abundance of available monitoring data and because the available IPPC-IED documentation, which is updated every 6–10 years, for most of the activities is dated 2017.

## Results and discussion

Following the methodology illustrated in the “Methods and materials” section, we first assessed the BPI for all pollution hotspots and then estimated the CBPI and the BQI along the river network. These latter indexes were estimated on the main watercourse only, neglecting the minor hydrographic network. This choice was driven by validation reasons: We focused on river networks where water quality monitoring stations are available; however, the methodology can also be applied to the minor network.

### Reno River

**Fig. 8:**
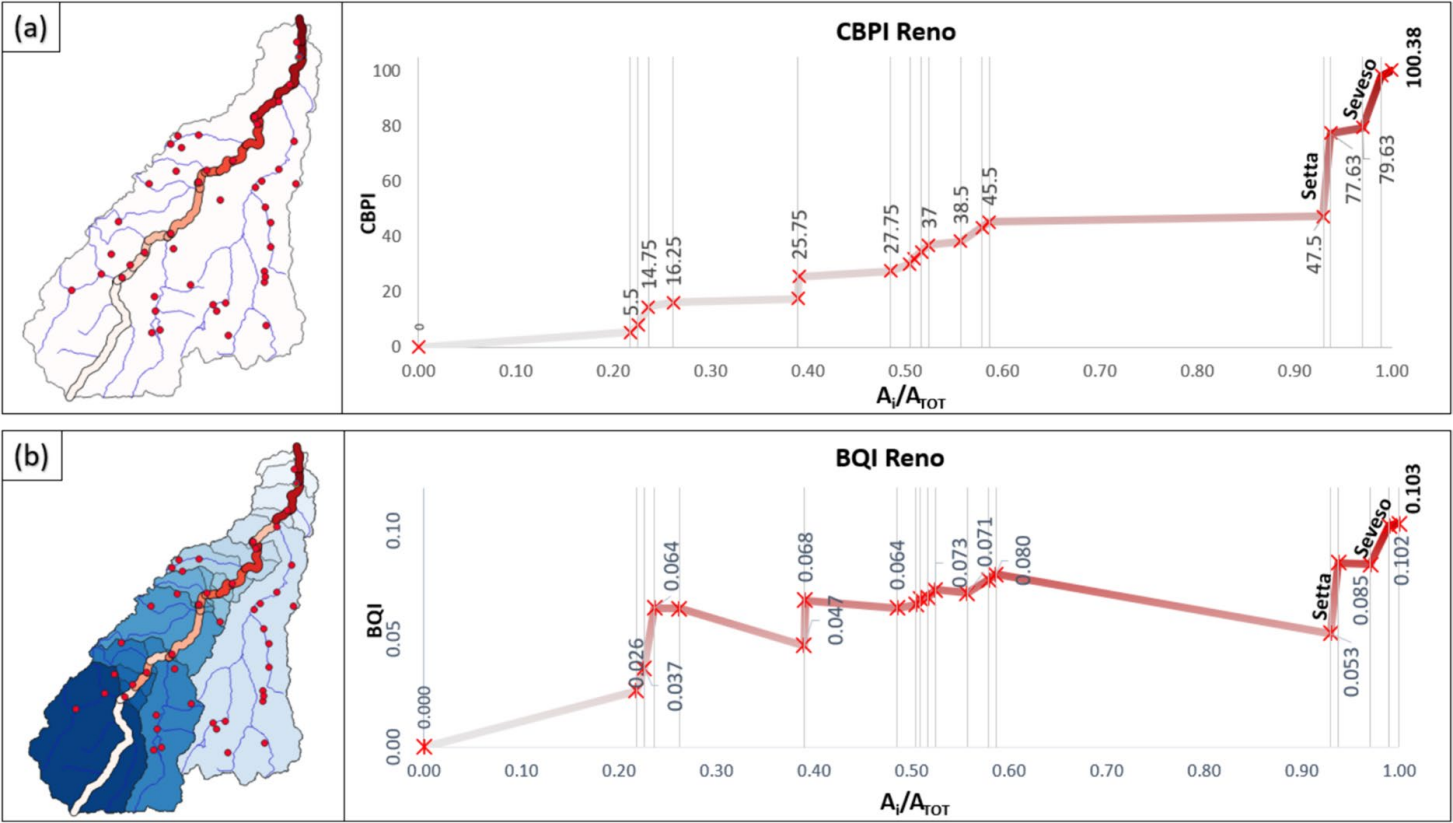
Spatial trend of CBPI (**a**) and BQI (**b**) in the Reno catchment

Referring to 46 sources on the basin, we identified the hydraulic paths, allocation points, and, hence, the river segments and subtended sub-basins (see the “Methods and materials” section). The cumulative (CBPI, Fig. 8a) and the biochemical quality (BQI, Fig. 8b) indexes were calculated for each river segment underlying the sub-basins (values of the indexes are shown as labels on both panels). The left boxes in Fig. 8 show source locations, hydraulic networks, and sub-basins identified according to the proposed methodology (see Fig. 4).

Vertical gray lines represent the discharge allocation points along the main river (see also Fig. 3). Being a cumulative index, the CBPI is strictly increasing moving downstream. Analyzing its values, a higher observed gradient indicates increased pressure from the industrial activities on the corresponding river segment. This representation facilitates a spatial assessment of the total loads entering the main river network due to hotspots’ weight and their geographical distribution in the basin.

Considering the BQI (Fig. 8b), decreasing trends can be observed moving downstream along the river in relation to the sub-basin extent of the segments and the pollution loads reaching the main river. In both panels, the chainage along the considered river network is represented in relative terms to the total river basin extent (A_i_/A_tot_) to enable a better comparison among the two indexes.

Examining both CBPI and BQI dynamics, it is evident that the most notable contribution, surpassing all other sources, originates from the Seveso plant, which discharges into the main river just before the basin outlet. The significant contribution labeled Setta is attributed to the confluence of a secondary river that collects a considerable set of pollutant sources into the main river network (see panels in Fig. 8).

**Fig. 9:**
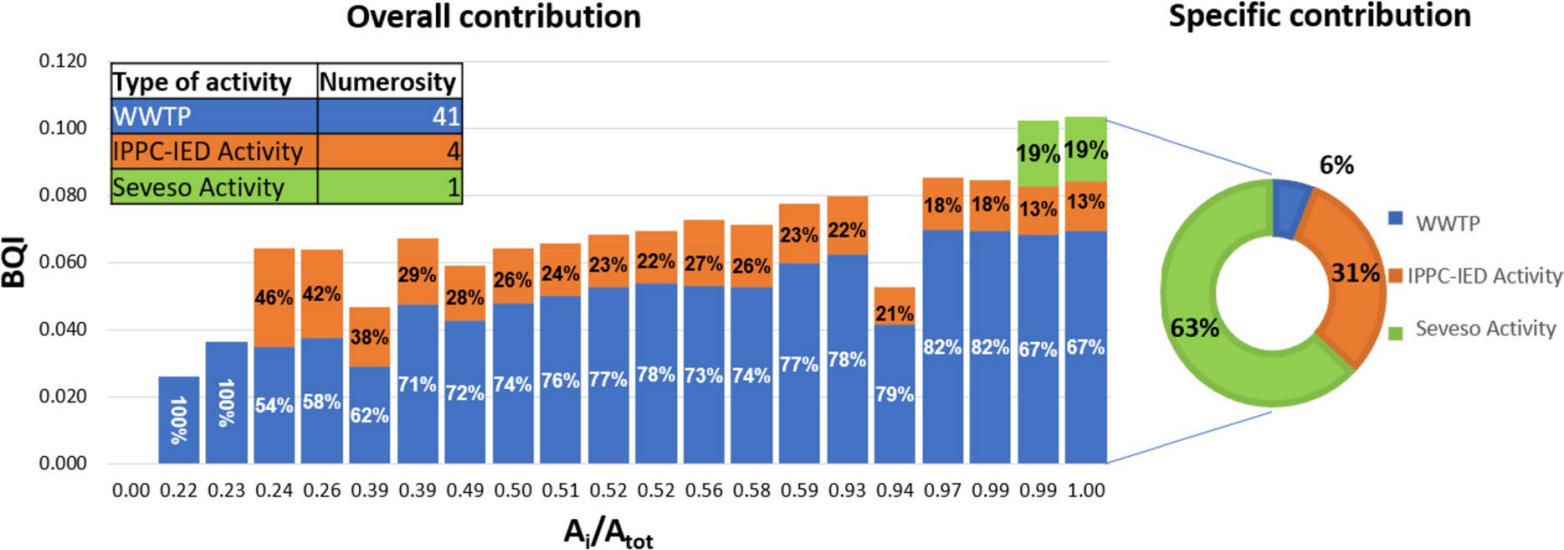
Overall (bars) and specific (pie plot) contribution on BQI values by type of activity (numbers of activities for category present in the study area are recalled in the table)

Figure 9 shows how different hotspot categories concours in defining the BQI. Specifically, the bars depict the overall contribution of various types of activity in reaching the index value along the main river network, with each bar corresponding to a specific river segment. Referring to the river outlet (last bar in Fig. 9), the greatest contribution (67%) is due to WWTPs in reason of their number (41 out of 46 considered sources), while small contributions are associated with IPPC-IED and Seveso activity: 14% and 19%, respectively. Referring to this value, the pie plot in Fig. 9 reports the specific contribution of each category, expressed as the ratio of the weight of each activity type to the number of plants present in the study area. The outcome highlights the significance of the Seveso plant, which emerges as particularly hazardous due to the pollutants involved in the production processes. On the contrary, although all WWTP plants together are responsible for 67% of the BQI, the weight of each of them is significantly smaller than the one associated with IPPC-IED and Seveso activities.

### Enza River

**Fig. 10:**
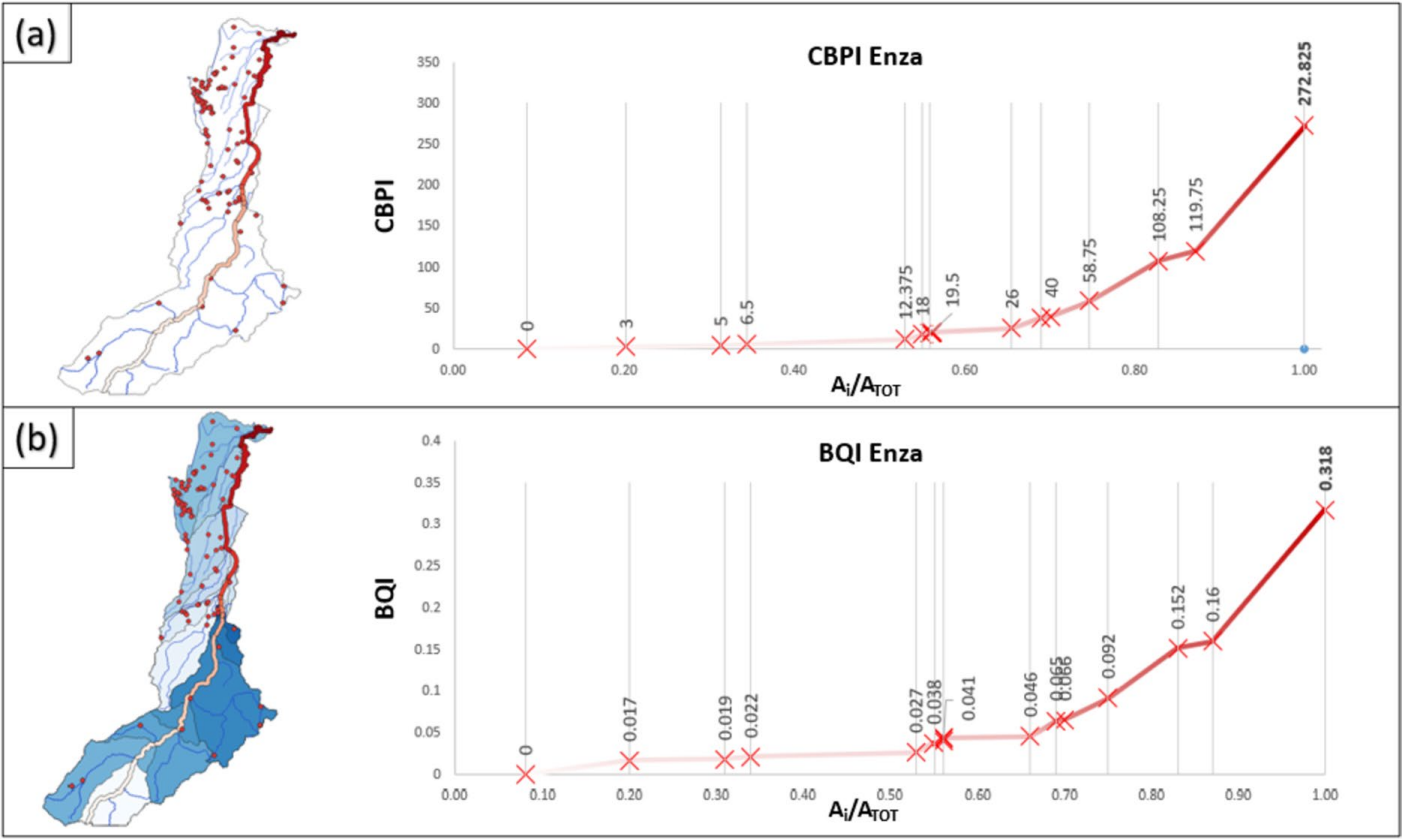
Spatial trend of CBPI (**a**) and BQI (**b**) in the Enza catchment

The Enza River basin comprises 96 pollution hotspots. The results shown in Fig. 10 reveal a consistent upward trend of both CBPI and BQI moving downstream. This trend reflects the distinct spatial distribution of industrial activities compared to the Reno River catchment, in which sources were numerous in the upstream part of the basin. In contrast, within the Enza catchment, the pollution hotspots are relatively scarce upstream and become more abundant moving downstream, resulting in the highest density of pollution sources. These urban hotspots contribute to the discharge at the basin outlet, where the maximum values of the indices are calculated.

**Fig. 11:**
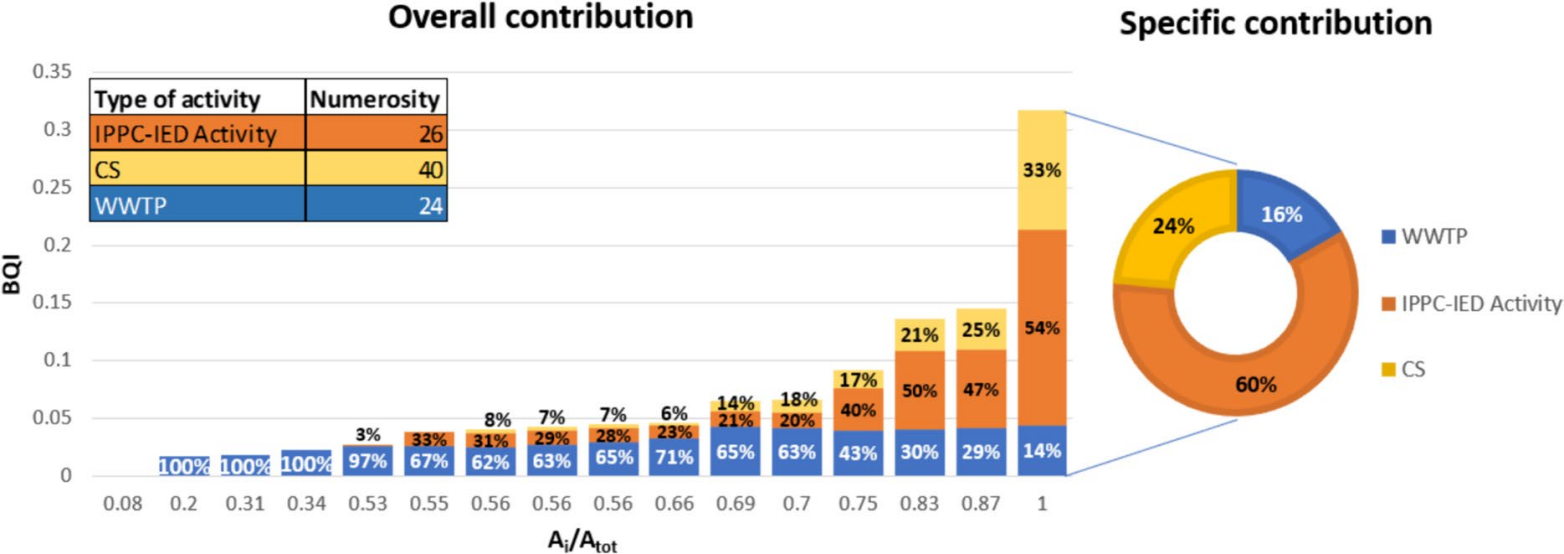
Overall (bars) and specific (pie plot) contribution on BQI values by type of activity (numbers of activities for category present in the study area are recalled in the table)

Analyzing the bar chart shown in Fig. 11, it is possible to observe the overall contribution of each activity: Examining, in particular, the last bar, the contaminated sites have a significant impact since their abundance in the final river segment that originates from the urban area of Parma. Analyzing the specific contribution of activities at the basin closure section, it becomes apparent that IPPC-IED activities make a highly significant contribution, accounting for 60% of the total.

### Parma River

**Fig. 12:**
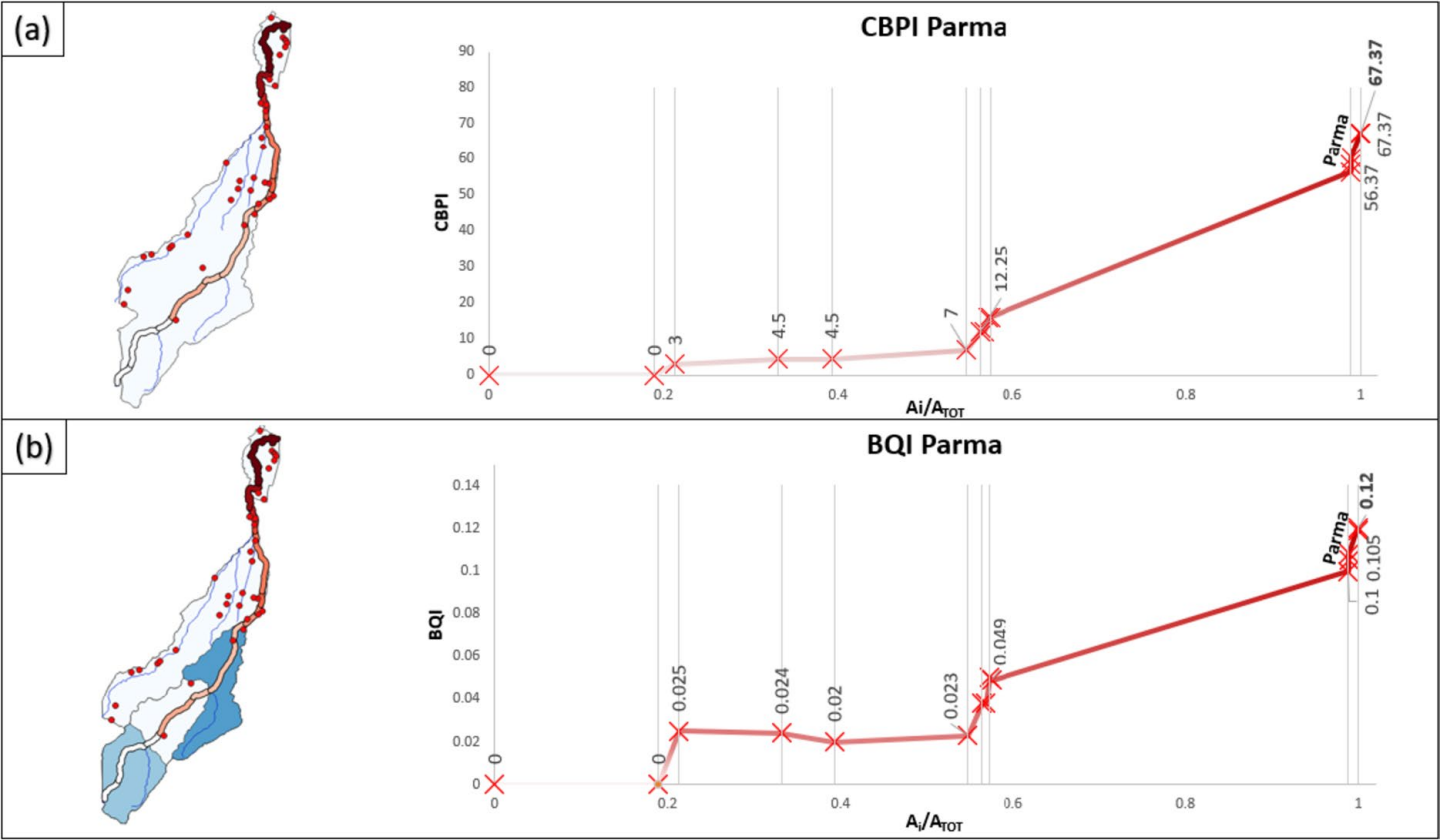
Spatial trend of CBPI (**a**) and BQI (**b**) in the Parma catchment

Looking at the CBPI in Fig. 12, it emerges that the Parma River suffers a lower pressure than those of other cases in reason of the limited number of activities in the study area. However, the value of BQI at the basin closure section is higher compared to that of the Reno River (0.103 for Reno and 0.119 for Parma). In fact, despite the overall lower pressures (CBPI), the smaller size of the Parma draining basin, here adopted as a proxy of pressure dilution and self-purification capacity, prevents the watercourse from reaching low BQI values. Therefore, the increase in the values of both indices occurs in the areas affected by the discharges from industrial activities in the river basin.

**Fig. 13:**
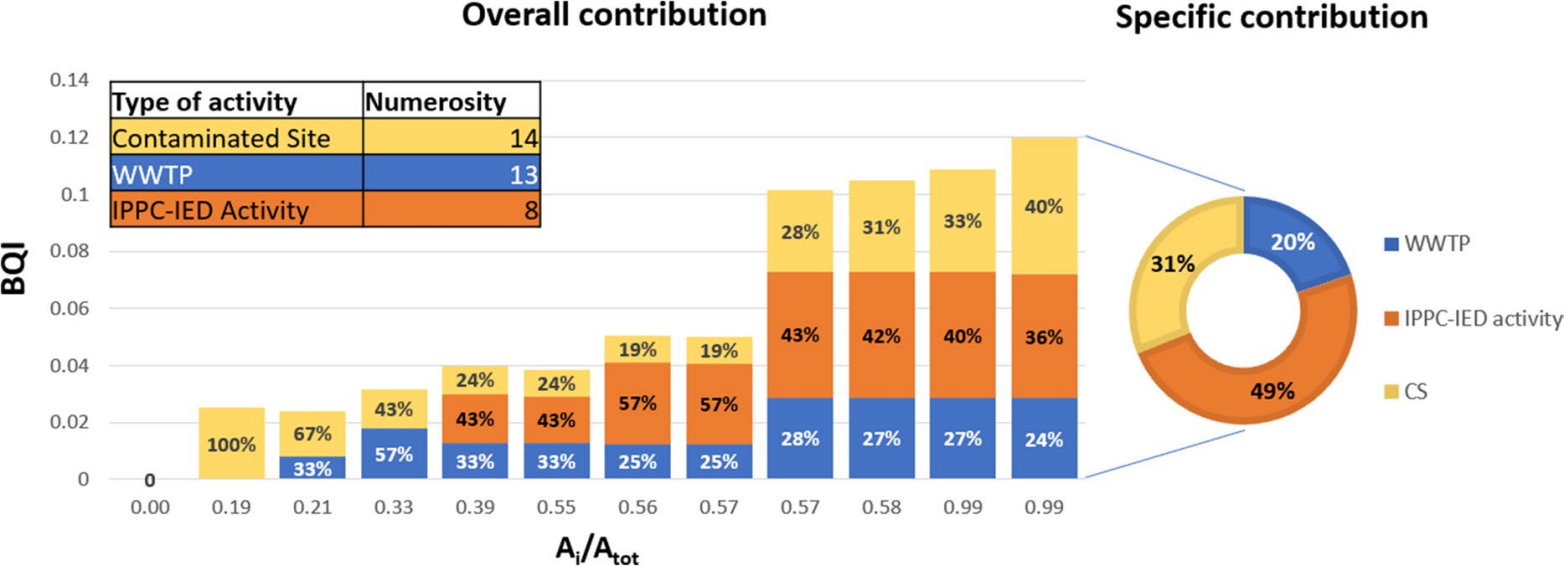
Overall (bars) and specific (pie plot) contribution on BQI values by type of activity (numbers of activities for category present in the study area are recalled in the table)

Analyzing the bar chart (Fig. 13), it can be observed that IPPC-IED activities make a significant and predominant contribution until reaching the discharges from the urban area of Parma, where contaminated sites become preponderant. However, even in this case, due to the intrinsic characteristics of this type of activity, IPPC-IED activities show a greater specific contribution.

### Validity check

A preliminary consistency check was first performed for BPI values. The idea behind this check is that, on average, Seveso activities are supposed to exert a bigger pressure on river bodies than IPPC-IED activities, which in turn should impact more than WWTP or contaminated sites (CS). Thus, the average BPI should follow the same order, regardless of the river basin in which activities are located. An analysis of 414 activities in the Emilia-Romagna region has shown that the average BPI follows the expected ranking, with Seveso activities scoring an average BPI of 5.86, IPPC-IED of 4.07, and WWTP and CS of 1.73 and 1.28, respectively.

Moreover, to further verify the overall validity of the proposed methodology, the BQI values have been compared with monitoring data related to the quality of freshwater available in the area of interest. These data have been taken from the ARPAE portal (the Emilia Romagna regional agency for prevention, environment, and energy; see the “Monitoring data for validation” section).

Special attention has been devoted to the yearly average of chemical oxygen demand (COD) recorded by monitoring stations in 2017, the reference year selected for the present study. The COD is a measure of the total oxygen demand, both via biodegradation or not, generated by pollution sources considered in the present study. Therefore, it can be regarded as closely correlated with overall water quality and the BQI.

It is worth mentioning that also dissolved oxygen (DO) could have been selected for the validity check; nevertheless, measured DO showed an almost negligible change along the considered rivers considered as a result of their good reaeration capacity, and thus, it could result in unsuitable for the spatial assessment of not be representative of the spatial variability of the water quality.

**Fig. 14:**
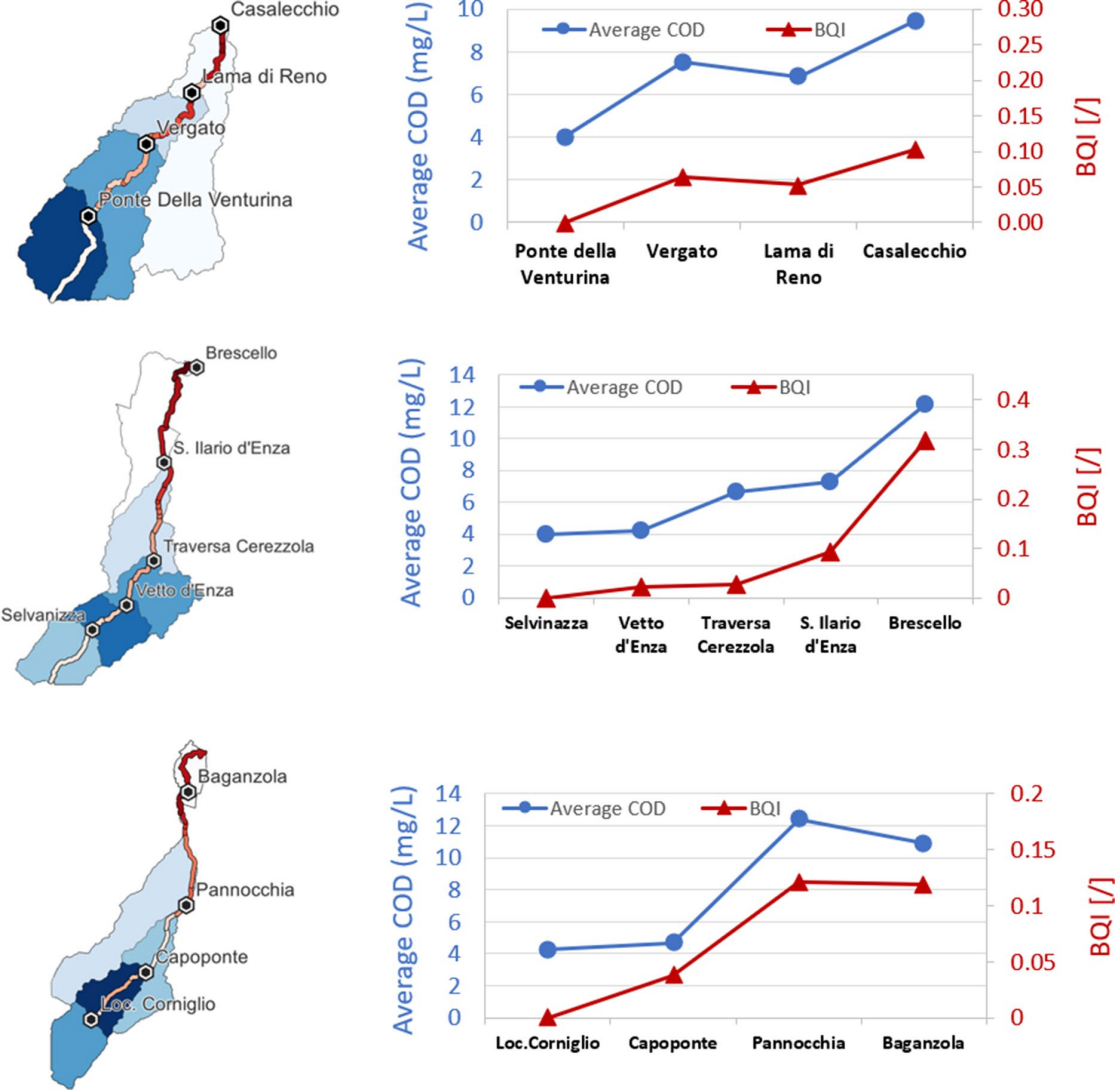
Comparison of recorded data (average COD) and the estimated BQI values for the three case studies (right); location of the gauging stations and BQI spatial variability are shown on the left

Regarding the Reno River, COD data was available at four stations (Fig. 14a). Since COD and BQI values are not directly comparable, the trend of the yearly average COD and BQI at monitoring stations is shown in Fig. 14a with two distinct axes. It can be observed that measured (COD) and predicted (BQI) water quality indexes are in agreement, with the latter effectively mirroring the COD dynamics along the watercourses. The same approach was followed for Enza and Parma Rivers. COD data were available at five (Fig. 14b) and four stations (Fig. 14c), respectively. Similarly to the previous application, the measured overall trend of water quality along both Enza (Fig. 14b) and Parma (Fig. 14c) rivers are well reproduced by BQI, which can be considered representative of the water status in the basins.

According to the results obtained, the proposed BPI is an expert-based and data-driven metric designed to evaluate the comprehensive biochemical load attributed to specific pollutant sources, such as industrial plants, wastewater treatment plants, contaminated sites, and others. The proposed approach is based on the attribution of a pressure index (BPI) to distinct types of anthropogenic activities based on objective and easily available data used to characterize industrial discharges. In this manner, it becomes feasible to perform a semi-quantitative assessment of biochemical pressures associated with a specific source in the aquatic environment (see. for example, bars and pie plots on Figs. 8 and 10).

A relevant added value of the proposed methodology is the possibility to spatially allocate the pressure index considering the total load distributed along the river and identifying the allocation points. This enables the identification of the comprehensive load expected at a specific river segment. In addition, the overall pressure across distinct rivers may be compared in terms of CBPI (Cumulative Biochemical Pressure Index).

Differently, the BQI index considers the size of the contributing area upstream of the allocation points, with the intent of accounting for hydrological characteristics of the basin (e.g., river flows and water permanence in the basin). In this context, the draining area serves as a proxy of natural processes which may contribute to mitigate the effects of anthropogenic pressures and define the water quality state. The BQI spatial variation gives a clear overview of the expected water quality along the watercourse, as well as the impact of significant point sources or industrial districts, such as production and industrial areas.

The reliability of the proposed methodology is proven by the validity check performed over three case studies where monitoring data were available. For all study areas, the increasing and decreasing trends of COD are well reflected in BQI values, which are therefore considered capable of capturing the spatial variability of biochemical water quality. This aspect may be significantly useful when planning measurement campaigns, detailed surveys to spotlight critical situations or to identify the best locations in view of the installation of additional gauging stations. While further investigations are necessary, it is evident that both CBPI and BQI present potential advantages over traditional monitoring networks in spatially evaluating river water quality.

Finally, it is worth noting that the BQI can account for temporal variability of the anthropogenic pressure, updating its value whenever the source documentation is updated (e.g., additional details on the production activity are provided) or additional pollution sources are detected. From this perspective, the methodology is also suitable as a tool for pre- and post-scenario evaluation, estimating the impact expected in the case of installation (or decommissioning) of new productive activities (or in case of changes in their characteristics).

While the methodology has demonstrated its validity, we acknowledge some limitations that need to be addressed: The first lies in the effort associated with consulting the documentation of the industrial activity, which constrains an extensive application to a wider scale; future developments will attempt to deal with such issues. A second weakness is related to the identification of the allocation point of pollutant sources (i.e., identification of the hydraulic paths). In this regard, the methodology adopted here relies on a DEM-based approach, which might produce misleading outcomes in flat or highly anthropized areas. In such regions, low slopes, the presence of ground depressions, infrastructure, and artificial drainage systems hinders the proper identification of hydraulic paths. In this respect, determining DEM topographic characteristics ensuring accurate path delineation cannot be established a priori. Thus, in flat areas, a supervised assessment is recommended.

Furthermore, the current approach only considers dilution capacity (using the draining areas as a proxy variable of the river flows) among overall self-purification processes (such as interaction with sediments, deposition, and adsorption), which description would entail the knowledge of several additional variable and river characteristics, as well as modeling solutions, that are not in line with the purpose of the study.

## Conclusions

This study introduces a novel short-cut methodology to assess the biochemical impact of human activities on freshwater bodies. The methodology was first theoretically conceived and then implemented and validated, referring to three case studies where monitoring data was available. Across all cases, the comparison confirmed the capability of the methodology to faithfully replicate variations in water quality along the river.

Methodological application relies on DEM and spatial allocation of the pollution sources, whose characteristics can be inferred from reports and documents typically available. Therefore, the BQI index serves as a reliable indicator of the current quality of freshwater. In situations where monitoring records are absent or limited in spatial and temporal coverage, they could serve as a proxy for estimating river water quality in relation to the anthropic activities present in the river basin.

Drawing from the outcomes of this preliminary investigation, we assert that the developed methodology exhibits promising potential as a supportive instrument to guide monitoring initiatives (e.g., at river segments where water quality is expected to be low) and to design local prevention measures along the hydrographic network, especially along minor watercourses where monitoring is usually scarce or totally missing. This approach intends to furnish supplementary knowledge to enhance the monitoring of river water quality, eventually assisting the definition of an efficient Programme of Measures (PoMs) required by the WFD.

## Data Availability

Most of the original data adopted for the methodology application are freely available in public repositories (links are reported in the text). On request, the corresponding author, Paola di Fluri, will provide processed data, as well as additional ones not available from the web, needed to support the findings of this study.

## Footnotes

1 Documentation retrieved consulting the website: https://www.arpae.it/it/arpae/amministrazione-trasparente/provvedimenti/provvedimenti-autorizzazioni-e-concessioni

2 Dataset available at the link: https://dati.arpae.it/dataset/rete-regionale-per-la-qualita-ambientale-acque-superficiali-fluviali-dati/resource/e5de6f16-96b6-47d9-ba94-bb77b3ce85b6
